# High Levels of S100A8/A9 Proteins Aggravate Ventilator-Induced Lung Injury via TLR4 Signaling

**DOI:** 10.1371/journal.pone.0068694

**Published:** 2013-07-18

**Authors:** Maria T. Kuipers, Thomas Vogl, Hamid Aslami, Geartsje Jongsma, Elske van den Berg, Alexander P. J. Vlaar, Joris J. T. H. Roelofs, Nicole P. Juffermans, Marcus J. Schultz, Tom van der Poll, Johannes Roth, Catharina W. Wieland

**Affiliations:** 1 Laboratory of Experimental Intensive Care and Anesthesiology, Academic Medical Center, Amsterdam, The Netherlands; 2 Center of Experimental and Molecular Medicine, Academic Medical Center, Amsterdam, The Netherlands; 3 Institute of Immunology, University of Münster, Münster, Germany; 4 Interdisciplinary Center of Clinical Research, University of Münster, Münster, Germany; 5 Pediatric Intensive Care Unit, Emma Children’s Hospital Academic Medical Center, Amsterdam, The Netherlands; 6 Department of Pathology, Academic Medical Center, Amsterdam, The Netherlands; 7 Department of Intensive Care, Academic Medical Center, Amsterdam, The Netherlands; 8 Center of Infection and Immunity, Academic Medical Center, Amsterdam, The Netherlands; University of Giessen Lung Center, Germany

## Abstract

**Background:**

Bacterial products add to mechanical ventilation in enhancing lung injury. The role of endogenous triggers of innate immunity herein is less well understood. S100A8/A9 proteins are released by phagocytes during inflammation. The present study investigates the role of S100A8/A9 proteins in ventilator-induced lung injury.

**Methods:**

Pulmonary S100A8/A9 levels were measured in samples obtained from patients with and without lung injury. Furthermore, wild-type and S100A9 knock-out mice, naive and with lipopolysaccharide-induced injured lungs, were randomized to 5 hours of spontaneously breathing or mechanical ventilation with low or high tidal volume (V_T_). In addition, healthy spontaneously breathing and high V_T_ ventilated mice received S100A8/A9, S100A8 or vehicle intratracheal. Furthermore, the role of Toll-like receptor 4 herein was investigated.

**Results:**

S100A8/A9 protein levels were elevated in patients and mice with lung injury. S100A8/A9 levels synergistically increased upon the lipopolysaccharide/high V_T_ MV double hit. Markers of alveolar barrier dysfunction, cytokine and chemokine levels, and histology scores were attenuated in S100A9 knockout mice undergoing the double-hit. Exogenous S100A8/A9 and S100A8 induced neutrophil influx in spontaneously breathing mice. In ventilated mice, these proteins clearly amplified inflammation: neutrophil influx, cytokine, and chemokine levels were increased compared to ventilated vehicle-treated mice. In contrast, administration of S100A8/A9 to ventilated Toll-like receptor 4 mutant mice did not augment inflammation.

**Conclusion:**

S100A8/A9 proteins increase during lung injury and contribute to inflammation induced by HV_T_ MV combined with lipopolysaccharide. In the absence of lipopolysaccharide, high levels of extracellular S100A8/A9 still amplify ventilator-induced lung injury via Toll-like receptor 4.

## Introduction

Acute lung injury (ALI) and its most severe form the acute respiratory distress syndrome (ARDS) is a devastating pulmonary condition with a high mortality rate, characterized by acute lung inflammation and edema [1]. Although mechanical ventilation (MV) is a lifesaving intervention in the management of these patients, it is well known that MV can contribute to the pathogenesis of ARDS [2]. Studies demonstrated that conventional MV could enhance but also initiate lung inflammation referred to as ventilator-induced lung injury (VILI) [2]–[4]. To date, low tidal volume (V_T_) MV is recommended. However, it has been reported that also MV with lung protective ventilator settings can cause (regional) hyperinflation and VILI [5]. The need for additional pharmacological interventions demands further research investigating potential new therapeutic targets.

Although the exact underlying mechanisms are incompletely elucidated, accumulating evidence indicates that mechanical stress and innate immunity pathways interact and compound lung injury [6]. One of the key findings in understanding the development of lung injury in the presence of MV was the discovery that microbial molecules, like lipopolysaccharide (LPS), have synergistic effects with MV in initiating or enhancing lung injury [7]–[9]. Innate immunity plays a central role in orchestrating pulmonary inflammation. Pathogen-associated molecular patterns are recognized by pattern recognition receptors and activation triggers an intense inflammatory response necessary to combat infection [10]. In the last decade it became clear that endogenous molecules released at sites of tissue injury, termed “alarmins” or “damage-associated molecular patterns” (DAMPs), also initiate and further activate innate immunity via the same receptors [10]. Of particular interest are S100A8 (also referred to as myeloid related protein 8) and S100A9 (also referred to as myeloid related protein 14) proteins, released into the extracellular space by activated phagocytes [11]. Indeed, neutrophils are among the first cells to infiltrate inflammatory regions and play a central role in the pathogenesis of ARDS and VILI [4]. The physiologically relevant form of S100A8 and S100A9 proteins is the S100A8/A9 complex in which S100A8 is thought to be the most active component [12]–[14]. S100A8/A9 levels correlate with disease activity in several inflammatory disorders, especially in rheumatoid arthritis, juvenile idiopathic arthritis, and inflammatory bowel disease [11]. Recently it became clear that these proteins are not only excellent biomarkers of inflammation, they also amplify the pro-inflammatory cascade via activation of innate immunity [12]; [15]. In lung tissue, up-regulation of S100A9 mRNA due to injurious MV was demonstrated [16]. Also, S100A8 and S100A9 proteins were detected in bronchoalveolar lavage fluid (BALF) of ARDS patients [17]–[19]. However, knowledge about the impact of S100A8/A9 proteins in inflamed ventilated lungs is limited.

We hypothesized that S100A8/A9 proteins are released during human and murine lung injury and contribute to the inflammatory response in a 2-hit model of LPS-induced lung injury combined with MV. In addition, we analyzed the effects of exogenous administered S100A8/A9 and S100A8 proteins in naïve mice, the impact of these proteins when combined with HV_T_ MV, and the role of TLR4 herein.

## Materials and Methods

### Patients

The study represents a secondary analysis of a previous prospective nested case control study where the relation between transfusion and the onset of ALI was investigated [20]. The Medical Ethics Committee from the University of Amsterdam approved the study protocol and written informed consent was obtained from all patients. Cardiac surgery patients were observed for the onset of ALI up to 30 hours after the surgical procedure. At onset of ALI a non-directed lung lavage was performed. Patients without ALI who were lavaged within 30 hours of ICU admission served as controls. In total 16 cases of ALI and 62 controls were identified. Study design and methods were described in detail previously [20].

In addition, to illustrate S100A8/A9 presence we stained paraffin-embedded lung biopsies for S100A9. Sections were obtained from 12 ICU patients during autopsy: 5 patients died without ALI and 7 patients died with ALI. Lung sections shown were from 2 patients: both were admitted to the ICU with an intracerebral bleeding, 1 patient developed ALI, the other patient succumbed without ALI.

### Mice

The Animal Care and Use Committee of the University of Amsterdam approved all experiments. Deficiency of the S100A8 gene in mice results in a lethal phenotype during embryogenesis [21]. Therefore S100A9 knockout (KO) mice, which lack both S100A9 and S100A8 proteins, despite normal S100A8 mRNA levels, were used [22]. It is thought that the turnover of isolated S100A8 is higher in the absence of its binding partner S100A9. Eight- to eleven week old S100A9 KO mice, generated as described previously [22] and backcrossed 10 times to a C57Bl/6 background, were bred in the animal facility of the Academic Medical Center of Amsterdam, The Netherlands. Age and sex matched wild-type (WT) mice were purchased from Harlan Sprague Dawley (Horst, The Netherlands) one week prior to the experiments and were also housed in the animal facility of the Academic Medical Center of Amsterdam.

### Experimental Groups

#### 1) WT versus S100A9 KO mice in (ventilator-induced) lung injury

Lungs of critically ill patients are exposed to diverse insults such as MV, infections, and systemic inflammation. These insults may interact and culminate in overwhelming lung inflammation often seen in ARDS patients. To study the role of S100A8/A9 proteins in these settings we used WT and S100A9 KO mice in a 1-hit and 2-hit lung injury model. Healthy animals and mice with pre-injured lungs, induced by 0.25 mg/kg lipopolysaccharide (LPS) (E. coli L4130, Sigma Aldrich) intranasally 1 hour before randomization, were assigned to a spontaneously breathing group (n = 6–8/group) or to a mechanically ventilated group (n = 6–8/group). Mice were ventilated with high (H)V_T_ or with a relatively low (L)V_T_ (see below MV strategy). After 5 hours all mice were sacrificed by exsanguination under general anesthesia. Lungs were used for histopathology, BALF, and lung tissue homogenates.

#### 2) Exogenous S100A8/A9 or S100A8 in vivo

To study the impact of extracellular S100A8/A9 or S100A8 proteins in the lung, healthy WT mice received recombinant mouse S100A8/A9 (30 µg/mouse) or S100A8 (30 µg/mouse) or vehicle (PBS) intratracheal. Mice were subsequently randomized to a spontaneously breathing group (n = 6) or to HV_T_ MV (n = 7). After 5 hours all mice were sacrificed by exsanguination under general anesthesia and lungs were used for BALF.

#### 3) Exogenous S100A8/A9 in TLR4 mutant mice

Next, we analyzed the role of TLR4 in pulmonary S100A8/A9 signaling. For this we used C3H/HeN mice (WT mice) and C3H/HeJ mice, that have homozygous mutated TLR4 genes leading to a TLR4 null phenotype. At start of 5 hours of HV_T_ MV, we administered S100A8/A9 proteins (30 µg/mouse) or vehicle (PBS) intratracheal to C3H/HeN mice (Charles River, Someren, the Netherlands) and C3H/HeJ mice (Jackson Laboratory, Bar Harbor, Maine). After 5 hours all mice were sacrificed by exsanguination under general anesthesia and lungs were used for BALF (n = 7–8/group).

#### Instrumentation and anesthesia

LPS, S100A8/A9, S100A8, or PBS were administered under 2–3% isoflurane anesthesia. In ventilated animals, a tracheotomy was performed and an Y–tube connector (1.0 mm outer diameter and 0.6 mm inner diameter, VBM Medizintechnik GmbH, Sulz am Neckar, Germany) was inserted into the trachea under general anesthesia with an intraperitoneal injection of KMA “induction”–mix: 7.5 µl per 10 gram of body weight of 1.26 ml 100 mg/ml ketamine, 0.2 ml 1 mg/ml medetomidine, and 1 ml 0.5 mg/ml atropine in 5 ml normal saline. Maintenance anesthesia consisted of 10 µl per 10 gram body weight of KMA “maintenance”-mix of 0.72 ml 100 mg/ml ketamine, 0.08 ml 1 mg/ml medetomidine and 0.3 ml 0.5 mg/ml atropine in 20 ml normal saline. Maintenance mix was administered via an intraperitoneal catheter (PE 10 tubing, BD, Breda, the Netherlands) hourly, every 30 minutes 0.2 ml sodium carbonate (200 mmol/l NaHCO_3_) was administered via the same intraperitoneal catheter throughout the experiment. Rectal temperature was maintained between 36.5–37.5°C using a warming pad. In a subset of the experiment heart rate and systolic blood pressure were non-invasively monitored using a murine tail-cuff system (ADInstruments, Spenbach, Germany). Directly after start of MV, after 2.5, and 5 hours of MV. Both remained stable throughout the experiment (See Data S1).

#### MV strategy

Methods of the murine MV-model used were published in detail previously [23]. Animals were placed in supine position and connected to a ventilator (Servo 900 C, Siemens, Sweden). During 5 hours mice were pressure controlled ventilated with an inspiratory pressure of 18 cm H_2_O (resulting in V_T_ ∼15 ml/kg (HV_T_)) or with an inspiratory pressure of 10 cm H_2_O (resulting in V_T_∼7.5 ml/kg (LV_T_)) under general anesthesia. Respiratory rate was set at 70 or 110 breaths per minute respectively, PEEP was set at 2 cm H_2_O, the fraction of inspired oxygen was kept at 0.5, and inspiration to expiration ratio was set at 1∶1. A sigh (sustained inflation with 30 cm H_2_O) for 5 breaths was performed hourly. After 5 hours of MV mice were sacrificed under general anesthesia by withdrawing blood from the carotid artery. This was used for blood gas analysis in a subset of the experiment, demonstrating adequate gas exchange in ventilated animals with no differences between WT and KO mice (See Data S2).

#### Purification of S100A8 and S100A8/A9

Murine S100A8 and S100A9 proteins were purified as described earlier for the human S100 proteins [24]. To obtain heterodimer complexes, purified homodimers were denatured in 8 M urea and mixed in equal amounts. Renaturation was allowed during extensive dialysis from acid pH to neutral pH in different steps. Protein identification was performed by electrospray ionization mass spectrometry. Possible endotoxin contaminations were eliminated by Endotrap column and quantified by limulus amebocyte lysate assay (BioWhittaker) and in blocking experiments using polymyxin B (Sigma). Limulus amebocyte assay did not detect LPS in the protein preparations (sensitivity ∼5 pg/µg protein).

#### Sampling

BALF was performed by instilling three times 0.5 ml of saline into the trachea. Cell counts were determined using a Coulter cell counter (Beckman Coulter, Fullerton, CA), differential cell counts were performed on cytospin preparations stained with Giemsa stain. Supernatant was stored at −20°C for further measurements. For histology, lungs were fixed in 4% formalin, embedded in paraffin, 4 µm sections were stained with hematoxylin–eosin and analyzed by a pathologist who was blinded for group identities. To score lung injury, 4 pathologic parameters were scored on a scale of 0–4: (a) oedema, (b) haemorrhage, (c) interstitial infiltration and (d) hyaline membranes [23]. Total histology score was expressed as the sum of the score for all parameters. In addition, S100A8 and S100A9 stainings were performed on lung sections as described previously [22]. Lung tissue homogenates were prepared by homogenizing lungs in 4 volumes of sterile 0.9% NaCl and these samples were subsequently lysed in 1∶2 lysis buffer containing 300 mM NaCl, 30 mM Tris, 2 mM MgCl_2_, 2 mM CaCl_2_, 1% Triton x-100 and Pepstatin A, Leupeptin and Aprotinin (all 20 ng/ml; pH 7.4). Homogenates were centrifuged and supernatants were stored at −20°C until further analysis.

#### Assays

Total protein levels were determined in BALF using a Bradford Protein Assay Kit (OZ Biosciences, Marseille, France). Interleukin (IL)–6, IL–1β, tumor necrosis–factor (TNF)–α, keratinocyte–derived chemokine (KC) and macrophage inflammatory protein (MIP)-2 levels were measured by enzyme–linked immunosorbent assay (R&D systems, Mineapolis, MN). Detection limits were 51 pg/ml for KC, IL-6, TNF-α and IL-1β. MIP-2 had a detection limit of 153 pg/ml. Immunoglobulin M (IgM) levels were analyzed as previously described [25]. S100A8/A9 concentrations were measured by sandwich enzyme–linked immunosorbent assay as previously described: human [26], mouse [14].

#### Statistical analysis

All data are presented as mean ± SEM. Two group comparisons were analyzed with a student t-test or Mann Whitney U-test depending on data distribution (ARDS versus no ARDS and WT versus KO). A secondary analysis compared WT mice of control, HV_T_ MV-only, LPS-only and HV_T_ MV/LPS groups. For this we used analysis of variance in conjunction with Bonferroni post hoc testing or a Kruskal-Wallis test with Mann-Whitney U-test, depending on data distribution. For the experiments were intratracheal vehicle was compared with S100A8/A9 or S100A8 protein exposure in naïve and ventilated mice we also used analysis of variance with Bonferroni post hoc analysis or a Kruskal-Wallis in conjunction with a Mann-Whitney U-test, depending on data distribution. All statistical analyses were carried out using GraphPad Prism version 5 (Graphpad Software; San Diego, CA). P values<0.05 were considered significant.

## Results

### S100A8/A9 Levels Increase in Clinical and Experimental Lung Injury

**Figure 1:**
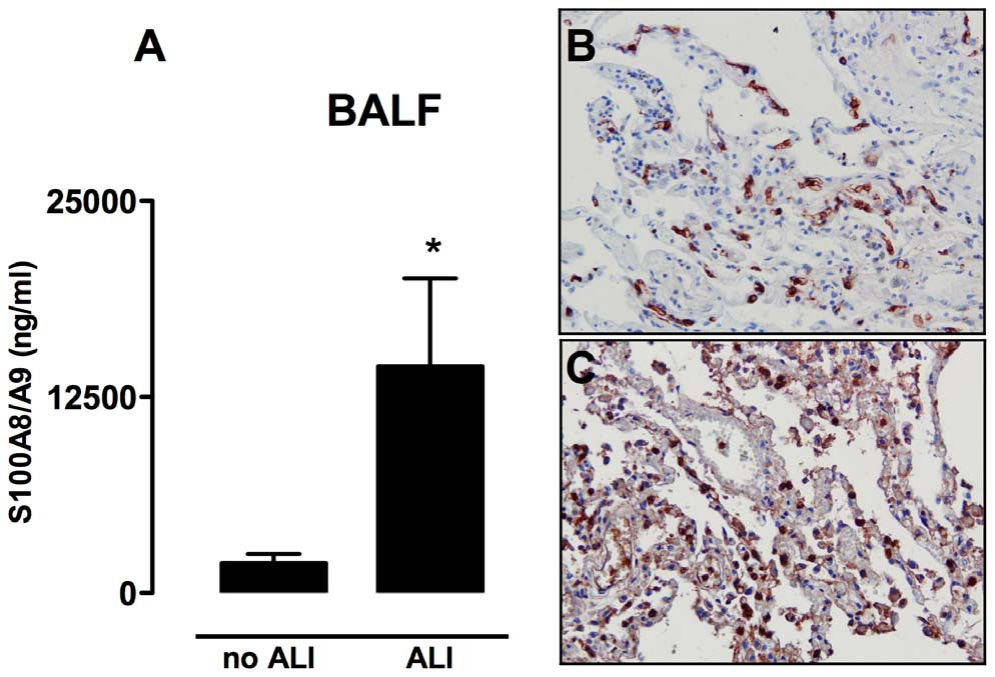
S100A8/A9 proteins increase in patients with mild ARDS. Presence of S100A8/A9 proteins in lung lavage fluid of patients with acute lung injury (ALI) (n = 16) and patients without ALI (n = 62) (A). Representative immunohistochemical stainings of S100A9 (staining in red, background in blue) of human lung tissue obtained from an ICU patient without ALI (B) and with ALI (C). Data are shown as mean ± SEM. *P<0.05.

In the prospective nested case control study, baseline characteristics of the cases (n = 16), and their controls (n = 62), were described in detail previously [20]. There were no differences in cardiac or pulmonary function between the groups pre-operatively. Patients who developed ALI were older, received more transfusions, and total operation time, clamptime, and pumptime were longer when compared to controls [20]. Also, PaO_2_/FiO_2_ ratios were reduced in ALI patients and several outcome parameters were also different: patients with ALI were mechanically ventilated longer and had a longer stay on the ICU and hospital (See Data S3) [20]. To determine whether levels of S100A8/A9 proteins increase during lung injury in these patients we analyzed S100A8/A9 concentrations in lavage samples. ALI patients had increased levels of S100A8/A9 proteins in BALF when compared to patients without ALI (fig. 1). In addition, we illustrated increased S100A9 presence in lung tissue of a patient who succumbed with ALI by immuno-histochemical staining, which was clearly more intense compared to an ICU patient who died without ALI (fig. 1b,c).

**Figure 2:**
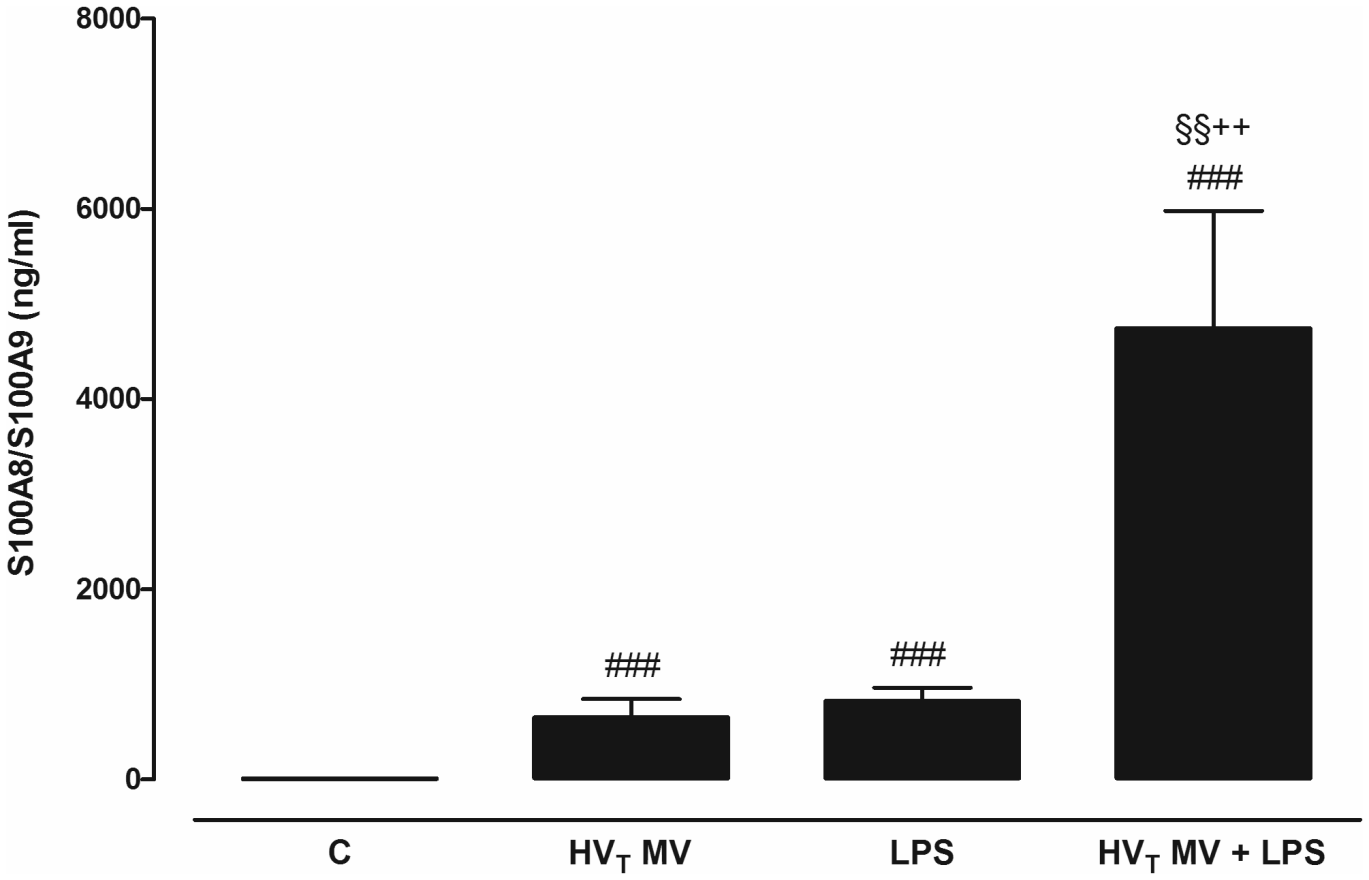
S100A8/A9 protein levels increase in mice with lung injury. S100A8/A9 levels in lung lavage fluid in a murine 2-hit lung injury model. Mice were spontaneously breathing (C), mechanically ventilated for 5 hours with high tidal volume (HV_T_ MV), received intranasal lipopolysaccharide (LPS; 0.25 mg/kg) followed by 5 hours spontaneously breathing (LPS), or received intranasal LPS followed by 5 hours of HV_T_ ventilation (HV_T_ MV+LPS). Data represent mean ± SEM of 8 mice per group. ^###^p<0.001 versus control, ^++^p<0.01 versus MV, ^§§^p<0.01 versus LPS.

**Figure 3:**
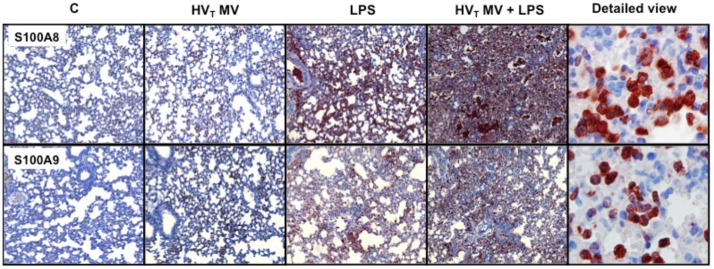
S100A8/A9 presence in lung tissue increases in mice with lung injury. Representative images of immunohistochemical stainings of S100A8 and S100A9 (specific staining in red, background staining in blue) of murine lung sections. Wild-type mice were spontaneously breathing (C), mechanically ventilated for 5 hours with high tidal volume (HV_T_ MV), received intranasal lipopolysaccharide (LPS; 0.25 mg/kg) followed by spontaneously breathing for 5 hours (LPS), or received intranasal LPS followed by 5 hours of HV_T_ mechanical ventilation (HV_T_ MV+LPS). Magnification 10×, detailed view of the HV_T_ MV+LPS group: magnification 100×.

In our mouse model, we found that both HV_T_ MV and LPS-induced injury resulted in significantly increased S100A8/A9 levels compared to non-ventilated control mice (fig. 2). The combination of both LPS and HV_T_ MV resulted in synergistically increased concentrations of S100A8/A9 in BALF; levels were higher when compared to non-ventilated controls, HV_T_ MV-only and LPS-only groups. To visualize S100A8 and S100A9 in the pulmonary compartment immuno-histochemical staining of mouse lung slides was performed. The expression of S100A8 and S100A9 increased with LPS administration or HV_T_ MV separately (fig. 3). In line with the S100A8/A9 protein levels in BALF, the most intense staining was seen in lung tissue of mice that received both HV_T_ MV and LPS. Higher magnification revealed that infiltrating neutrophils were the main S100A8 and S100A9-expressing cells (fig. 3). Healthy S100A9 KO mice lack both S100A9 and S100A8 proteins and thus biologically active S100A8/A9 heterodimers in myeloid cells [19]. For additional control purposes we also measured BALF S100A8 levels and stained lung tissue for S100A8 in S100A9 KO mice undergoing the 1 or 2-hit injury. S100A8 could not be detected in BALF. Moreover, in contrast to WT mice, no increased S100A8 staining was seen in KO mice undergoing the 1 or 2-hit injury (see Data S4).

### S100A9 Deficient Mice are Partially Protected in a HV_T_ MV/LPS 2-hit Lung Injury Model

**Figure 4:**
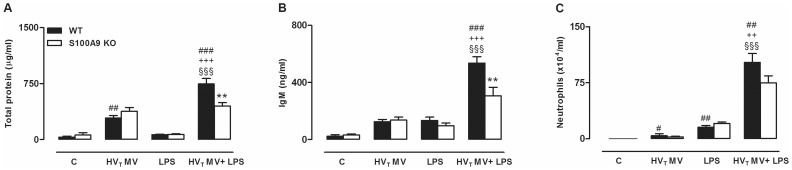
Barrier dysfunction is attenuated in S100A9 knockout mice undergoing a 2-hit lung injury model. Total protein levels (A), immunoglobulin M (IgM) content (B) and neutrophil counts (C) in bronchalveolar lavage fluid of wild-type (WT) and S100A9 knockout (KO) mice. Animals were spontaneously breathing (C), high tidal mechanically ventilated (HV_T_ MV), exposed to LPS followed by spontaneously breathing (LPS) or exposed to LPS followed by HV_T_ mechanical ventilation (HV_T_ MV+LPS). Data represent means (SEM) of 6–8 mice per group. **p<0.01 WT versus KO, ^###^p<0.001, ^##^p<0.01, ^#^p<0.01 versus WT C. ^§§§^p<0.001 versus LPS-only. ^+++^p<0.001, ^++^p<0.01 versus HV_T_ MV-only.

**Figure 5:**
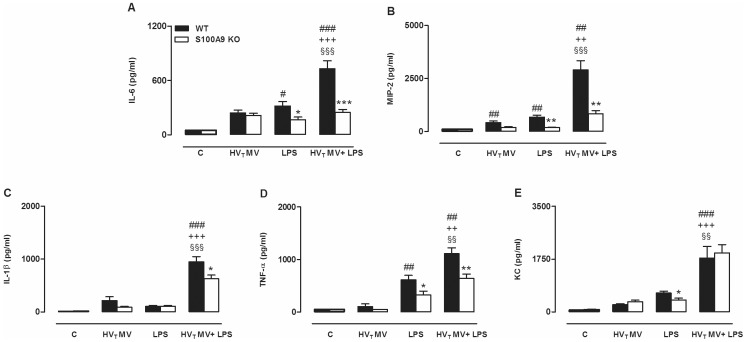
Inflammation is attenuated in S100A9 knockout mice undergoing a 2-hit lung injury model. Cytokine and chemokine concentrations in lung lavage fluid of wild-type (WT) and S100A9 knockout (KO) mice. Animals were spontaneously breathing (C), high tidal mechanically ventilated (HV_T_ MV), exposed to LPS followed by spontaneously breathing (LPS), or exposed to LPS followed by HV_T_ mechanical ventilation (HV_T_ MV+LPS). Levels of interleukin (IL)–6 (A), macrophage inflammatory protein (MIP)-2 (B), tumor necrosis factor-α (TNF-α) (C), IL-1β (D), and keratinocyte–derived chemokine (KC) (E) were determined. Data represent means (SEM) of 6–8 mice per group. *p<0.05, **p<0.01, ***p<0.001 KO versus WT mice, ^###^p<0.001, ^##^p<0.01, and ^#^p<0.05 versus WT C, ^§§§^p<0.001 and ^§§^p<0.01 versus LPS-only, ^+++^p<0.001, and ^++^p<0.01 versus HV_T_ MV-only.

**Figure 6:**
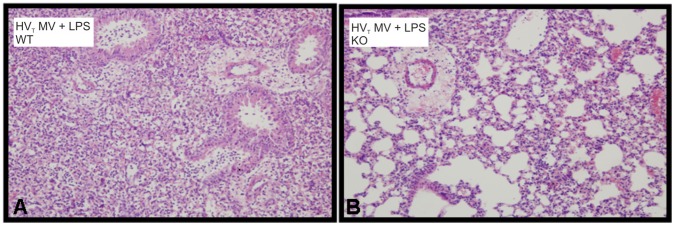
Histopathological changes were reduced in S100A9 knockout mice undergoing 2-hit lung injury. Representative histological sections of hematoxylin and eosin stained lungs of a wild-type (WT) mouse (A) and a S100A9 knockout (KO) (B) mouse exposed to lipopolysaccharide (LPS) and high tidal volume mechanical ventilation (HV_T_ MV). Original magnification x20 (in set x40).

To determine if the increased presence of S100A8/A9 proteins influenced lung injury and inflammation, we compared WT with S100A9 KO mice. HV_T_ MV-only and LPS-only both induced lung injury and the HV_T_ MV/LPS double-hit boosted lung injury: total protein levels, IgM concentrations, and neutrophil influx into the alveolar compartment were significantly increased when compared to control, HV_T_ MV-only, and LPS-only groups (fig. 4). No differences between S100A9 KO and WT mice were found in the control, HV_T_ MV-only and LPS-only groups. However, S100A9 KO mice undergoing the double hit demonstrated attenuated alveolar-epithelial permeability when compared to WT mice. This was demonstrated by a lower total protein content and IgM concentration in BALF. The same trend was seen for neutrophil influx although this did not reach statistical significance. To further analyze the lung inflammatory response in S100A9 KO mice, concentrations of cytokines and chemokines were measured in BALF. In line with the lung permeability measurements, inflammation was most severe in mice that were exposed to both LPS and overinflation. HV_T_ MV/LPS double hit increased the concentrations of BALF IL-6, MIP-2, Il-1β, TNF-α, and KC compared to the control, HV_T_ MV-only and LPS-only groups (fig. 5). As compared to WT mice, S100A9 KO mice had reduced levels of IL-6, MIP-2, IL-1β and TNF-α after both LPS and HV_T_ MV (fig. 5). Furthermore, inflammation was also attenuated in the LPS-only group demonstrated by lower IL-6, KC, MIP-2, and TNF-α concentrations compared to WT mice. Again, no significant differences were found in cytokine concentrations of WT and KO mice in the control group, and the HV_T_ MV-only group. In addition, we analyzed inflammation in lung parenchyma (See Data S5). In line with levels in BALF, IL-6, MIP-2 and TNF-α levels were significantly lower in lung tissue homogenates of S100A9 KO mice compared to WT mice of the HV_T_ MV/LPS group. The difference between WT and KO mice of the LPS group was less prominent in lung parenchyma, only TNF-α concentrations were reduced in S100A9 KO mice. Next, we analyzed lung histopathology slides. Whereas no significant differences in histopathology scores were found between WT and KO mice of control, HV_T_ MV-only, and LPS-only groups (table 1), histopathology scores of S100A9 KO mice undergoing the double hit were attenuated when compared to WT mice (table 1, fig. 6).

**Table 1:** Histopathology scores.

Group		WT		S100A9 KO	
**C**	0.50 [0.3]		1.50 [0.4]		
**HV_T_ MV**	2.33 [0.3]		2.67 [0.4]		
**LPS**	2.50 [0.6]		2.14 [0.4]		
**HV_T_ MV+LPS**	6.33 [0.61]		4.43 [0.5]*		

Lung injury scores of wild-type (WT) and S100A9 knockout (KO) mice of the non-ventilated control group (C), high tidal mechanically ventilated group (HV_T_ MV), LPS-exposed non-ventilated group (LPS), and LPS-exposed followed by mechanical ventilation group (HV_T_ MV+LPS). Data represent mean ± [SEM] of n = 6–7 mice/group.

* p<0.05 compared to WT mice of the HV_T_ MV+LPS group.

### S100A8/A9 Proteins in LV_T_ MV

After we have demonstrated that deficiency of S100A8/A9 reduces lung injury in overinflated inflamed lungs we analyzed the role of these proteins during LV_T_ MV. Five hours of LV_T_ MV induced S100A8/A9 proteins in BALF (See Data S6). Levels were higher in LPS-exposed LV_T_ MV ventilated mice. Again, we analyzed total protein, IgM, neutrophil influx, and cytokines and chemokines in BALF. In mice with healthy lungs and also in animals with inflamed lungs, induced by LPS-inhalation, we observed no differences between WT and KO mice in these measures of injury and inflammation after 5 hours of LV_T_ MV (table 2). These data indicate that S100A8/A9 proteins are less important in non-overstretched lung areas.

**Table 2:** S100A8/A9 in non-overstretched lung areas.

	Healthy				LPS-exposed			
	LV_T_ WT		LV_T_ KO		LV_T_ WT		LV_T_ KO	
**Total protein (µg/ml)**	85 [17]		64 [17]		131 [15]		164 [12]	
**IgM (ng/ml)**	58 [13]		30 [5]		107 [11]		126 [18]	
**Neutrophils (×10^4^/ml)**	4.1 [1.8]		2.7 [0.8]		25 [2.9]		25 [2.9]	
**IL-6 (pg/ml)**	206 [30]	204 [38]		532 [56]		620 [63]		
**KC (pg/ml)**	135 [26]	189 [35]		1382 [342]		1767 [518]		
**MIP-2 (pg/ml)**	81 [17]	57 [19]		1436 [439]		1093 [266]		
**IL-1β (pg/ml)**	62 [8]	57 [18]		152 [9]		201 [35]		
**TNF-α (pg/ml)**	B.D.	B.D.		614 [64]		386 [77]		

Total protein, immunoglobin M (IgM), neutrophil influx, cytokines and chemokines in bronchoalveolar lavage fluid of wild-type (WT) and S100A9 knockout (KO) mice. Animals with healthy lungs or with pre-existing lung injury induced by lipopolysaccharide exposure were ventilated for 5 hours with low tidal volumes (LV_T_) (∼7.5 ml/kg). Data represent mean (SEM) of n = 6–8 mice/group. Below detection limit: B.D.

### Exogenous S100A8/A9 Proteins Induce Mild Lung Inflammation in Healthy Mice

**Figure 7:**
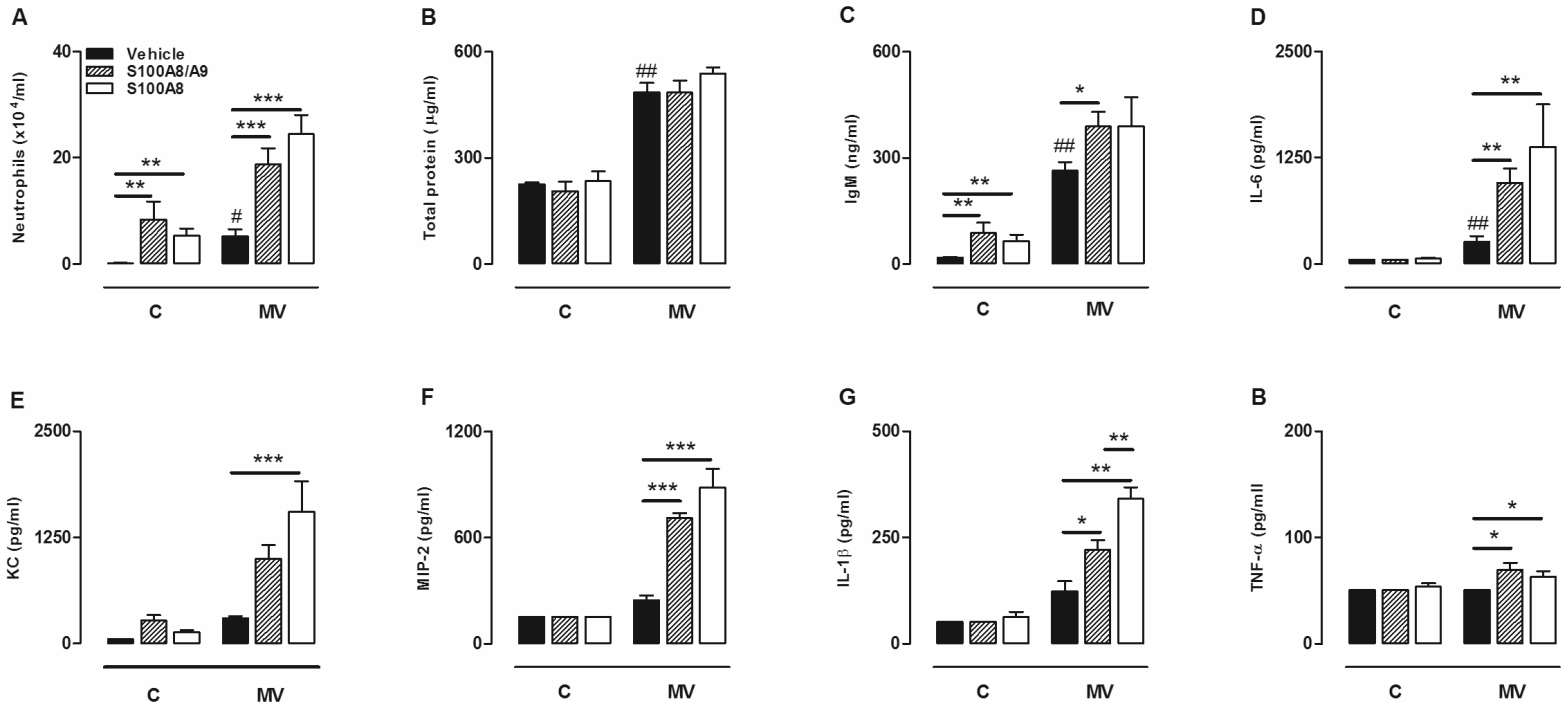
Exogenous S100A8/A9 proteins amplify MV-induced lung inflammation. Neutrophil influx (A), Total protein (B), immunoglobulin M (IgM) (C), interleukin (IL)-6 (D), keratinocyte-derived chemokine (KC) (E), macrophage inflammatory protein (MIP)-2 (F), IL-1β (G) and tumor necrosis factor (TNF)-α concentrations (H). Mice received S100A8/A9 (30 µg/mouse) or S100A8 (30 µg/mouse) or vehicle intra tracheal at start of five hours of spontaneously breathing (C) or high tidal volume mechanical ventilation (MV). Data represent mean (SEM) of 5–7 mice per group. *p<0.05, **p<0.01, ***p<0.001 versus vehicle treated mice. +p<0.05 S100A8 versus S100A8/A9 treated mice. ^#^p<0.05, ^##^p<0.01 ventilated vehicle treated versus control vehicle treated mice.

In a second set of experiments we analyzed if S100A8/A9 proteins could elicit inflammation in otherwise healthy lungs. Within 5 hours, S100A8/A9 and S100A8 proteins induced neutrophil recruitment into the alveolar compartment (fig. 7). Total protein levels were not affected by S100A8/A9 or S100A8 protein instillation but BALF IgM levels were elevated. Cytokines and chemokines were not significantly affected. Additionally, no differences were detected between S100A8/A9 and S100A8 instillation.

### S100A8/A9 Proteins have Synergistic Effects with Mechanical Stress

To determine if S100A8/A9 have additive effects during VILI in the absence of LPS, we administered these proteins also to naive mice and ventilated them with the HV_T_ MV strategy. S100A8/A9 and S100A8 exposure both resulted in significantly more neutrophil influx into the alveolar compartment compared to vehicle-treated mice (fig. 7). Total protein levels were not influenced by administration of S100 proteins. IgM levels however, tended to be higher reaching significance for S100A8/A9 exposed mice. Concentrations of the inflammatory mediators IL-6, IL-1β, MIP-2, and TNF-α were significantly increased in BALF due to exogenous S100A8/A9 and S100A8 compared to vehicle treated ventilated mice. S100A8-exposed ventilated mice tended towards more inflammation which was significant for the IL-1β levels. These experiments suggest that when pulmonary S100A8/A9 or S100A8 levels are highly increased, these proteins have additive effects in overinflated lung areas in enhancing pulmonary inflammation.

### S100A8/A9 Proteins Aggravate VILI via TLR4 Signaling

**Figure 8:**
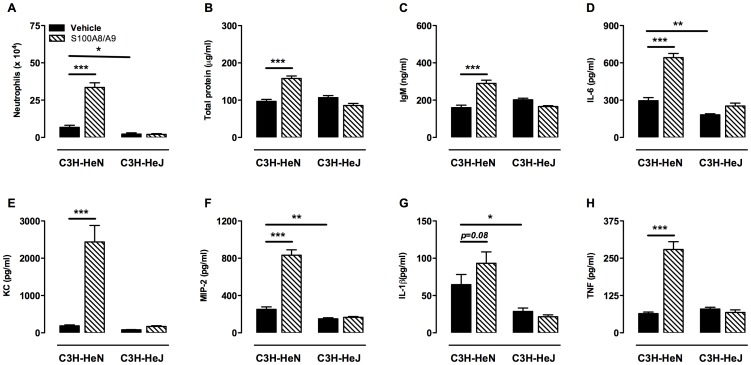
Exogenous S100A8/A9 proteins amplify VILI via Toll-like receptor 4. Total protein (A), immunoglobulin M (IgM) (B) Neutrophil influx (C), interleukin (IL)-6 (D), keratinocyt-derived chemokine (KC) (E), macrophage inflammatory protein (MIP)-2 (F), IL-1β (G) and tumor necrosis factor (TNF)-α concentrations (H) in C3H-HeN and C3H-HeJ mice exposed to S100A8/A9 (30 µg/mouse) or vehicle intratracheally at start of high tidal volume mechanical ventilation. Mice were ventilated for 5 hours. Data represent mean (SEM) of 7–8 mice per group. *p<0.05, **p<0.01, ***p<0.001 versus vehicle treated mice. ^#^p<0.05 S100A8 versus S100A8/A9 treated mice.

To evaluate if TLR4 signaling was required for S100A8/A9-induced aggravation of VILI we analyzed pulmonary inflammation in TLR4 mutant mice. First, we tested if presence of S100A8/A9 also aggravates HV_T_ MV-induced inflammation in the C3H mouse strain. We observed, in line with the results above, significantly more neutrophils in the alveolar compartment in ventilated S100A8/A9 exposed C3H/HeN mice compared to vehicle exposed animals (fig. 8). Also, total protein, IgM, IL-6, IL-1β, MIP-2, KC, and TNF-α levels were increased in BALF by S100A8/A9 exposure. Strikingly, S100A8/A9 administration to ventilated C3H/HeJ mice did not affect any of the inflammatory parameters compared to vehicle exposed C3H/HeJ mice (fig. 8). These data clearly reveal that the TLR4 pathway is required for S100A8/A9 proteins to aggravate VILI. Noteworthy, we also found differences between neutrophil influx and cytokine and chemokine levels when comparing ventilated vehicle exposed C3H/HeN with C3H/HeJ mice which again underscores the importance of TLR4 signaling in VILI.

## Discussion

The excessive pulmonary inflammatory response in ALI/ARDS is complex and knowledge about key modulators is limited. Here we reported that S100A8/A9 is an important DAMP able to amplify pulmonary inflammation during HV_T_ MV: (1) S100A8/A9 proteins were increased in lungs of ALI patients and in mice with LPS- or MV-induced lung injury, (2) S100A8/A9 levels were synergistically increased upon HV_T_ MV/LPS double hit and targeted deletion of S100A9 in this group attenuated pulmonary permeability and inflammation, (3) in gain of function experiments extracellular S100A8/A9 induced mild neutrophil influx in healthy recipients, and (4) in the absence of LPS S100A8/A9 clearly aggravated VILI via TLR4 dependent mechanisms.

The tidal volumes used in our mouse model are higher then those normally used in clinical practice. However, although the use of low tidal volume ventilation (6 ml/kg body weight) in ARDS is widely accepted, a recent multicenter multinational study indicated that higher tidal volumes (>8 ml/kg body weight) in patients with evidence for ARDS are still used [27]. More importantly, computed tomography studies demonstrated that during ARDS lungs are non-uniformly expanded [28]. Extensive areas are fluid filled and collapsed resulting in a reduced effective alveolar volume. Even the use of low tidal volume ventilation can lead to hyperinflation in less-affected lung areas, indicating that VILI is a regional phenomenon [5]. Studies on the effect of hyperinflation are therefore still relevant to reveal information on lung injury caused by MV, as hyperinflation is an important factor contributing to the development of VILI.

S100A8/A9 proteins are highly expressed in neutrophils, representing 40% of the cytosolic protein content [11]. These proteins are also found in monocytes, early differentiation stages of macrophages, and can be induced under inflammatory conditions in keratinocytes and epithelial cells [11]. Thirty minutes of injurious MV in rats resulted in pulmonary S100A9 mRNA up-regulation [16] and LPS stimulation of bronchial epithelial cells *in vitro* increased S100A8 and S100A9 protein release [29]. In addition, we here report that S100A8/A9 protein levels in BALF also elevate during an *in vivo* murine model of VILI and LPS-exposure. Our human data demonstrate high pulmonary S100A8/A9 levels in ICU patients suffering from ALI, which is in line with previous findings [17]–[19]. Although these results cannot be directly linked to our animal experiments since the settings that led to lung injury development were not similar, our patient study does illustrate once more that human lung injury is associated with a local rise in S100A8/A9 levels [17]–[19].

Studies in mice without pre-existing lung injury showed that VILI is in part dependent on TLR4 and the inflammasome [30]–[33]. It is currently unclear how TLR4 signaling is activated during VILI but evidence suggests an important role for endogenous ligands [30]. MV can result in the release of TLR4 activating DAMPs such as hyaluronan, high mobility group box-1, and heat shock proteins [34]. In addition, we observed that S100A8/A9 is released during MV. However, elimination of only S100A8/A9 had no effect on MV-induced VILI. In contrast, LPS-induced lung injury resulted in differences between WT and KO mice in reduction of several inflammatory parameters. IL-6, KC, MIP-2, and TNF-α in BALF were lower in KO mice, consistent with a current view that S100A8/A9 amplifies LPS-induced TLR4 activation [14].

The current theory for development of ARDS suggests a 2-hit mechanism [4]. The lung can be ‘primed’ by a direct insult such as pneumonia, aspiration, major surgery, and trauma, which sets the balance for an increased inflammatory response to a second insult such as MV. Previous experimental studies and the results presented in this manuscript indeed show synergistic interactions between innate immunity and MV: in the presence of microbial products the inflammatory response towards HV_T_ MV is enhanced [7]–[9]. Presence of the DAMP S100A8/A9 highly increased upon the HV_T_ MV/LPS double hit. We demonstrated here that these proteins are not only a marker of increased lung damage, they contributed to injury and inflammation in this 2-hit setting.

A hallmark of ARDS is loss of alveolar-capillary membrane barrier function resulting in increased vascular permeability [1]. A previous *in vitro* study reported S100A8/A9 proteins to induce endothelial disintegration and have cytotoxic effects contributing to endothelial damage [35]. In line, we observed reduced alveolar-capillary membrane permeability in S100A9 deficient mice, demonstrated by lower total protein content and IgM levels in BALF. S100A9KO mice undergoing the HV_T_ MV/LPS double hit also demonstrated attenuated histopathological changes and lower cytokine and chemokine levels in BALF and lung tissue compared to WT mice.

To further characterize the extracellular contribution of these proteins in lung inflammation we administered exogenous proteins to healthy animals. *In vitro* it was previously demonstrated that S100A8 alone, in the absence of LPS, was capable of inducing TNF-α expression in bone marrow cells [14]. The S100A8/A9 complex had only additive effects in combination with LPS stimulation. Our *in vivo* experiments demonstrate that S100A8/A9 and S100A8 alone induce neutrophil recruitment in lungs of naive mice. These data are supported by other *in vivo* experiments where chemotactic features of the S100A8/A9 complex and S100A8 were shown in an arthritis air pouch model in mice [36]–[37]. In LPS-models however, the administration of S100A8/A9 proteins has led to conflicting results. It has been demonstrated that S100A8/A9 and S100A8 promote LPS-induced shock in mice [14], but a protective effect of extracellular S100A8/A9 on LPS-induced liver damage has also been reported [38]. Very recently it was shown that S100A8 administration attenuated inflammation and injury in a mouse model of endotoxemia [39]. These results suggest that S100A8/A9 proteins might have a dual role in inflammation depending on experimental setting (extent of inflammation, time or dosis of S100 exposure etc.). Our experiments demonstrated that in the absence of LPS, high levels of S100A8/A9 and S100A8 have synergistic effects with HV_T_ MV and markedly enhance VILI. This provides evidence that the interaction between MV and innate immunity is not restricted for bacterial products and that in the inflamed lung, in the absence of infection, endogenous stimuli also amplify the inflammatory response to HV_T_ MV.

In line with prior reports we observed that VILI is attenuated in TLR4 mutant mice [30]; [31]; [40]. Using TLR4 deficient mice it was previously clearly demonstrated that TLR4 mediates VILI in healthy animals [30]–[31] and also inflammation in HV_T_ ventilated LPS challenged mice [40]. This manuscript also demonstrates that TLR4 plays a crucial role in S100A8/A9 induced aggravation of VILI, underscoring again the importance of this innate immune receptor in VILI. Animal studies have shown that MV increases the expression of pulmonary TLR4 [30]; [41]. It can be speculated that the up-regulated expression of TLR4 in overstretched lung tissue makes the lung more sensitive for high levels of S100A8/A9 proteins.

In a murine heart failure model it was demonstrated that S100A8/A9 proteins also activate the receptor for advanced glycation end products (RAGE) [15]. RAGE is an innate immune receptor that recognizes multiple ligands, including DAMPs like S100A8/A9, S100A12, and high mobility group box-1 [42]. RAGE is highly expressed by human and murine lung tissue and therefore an interesting receptor for future VILI research. In this manuscript we did not study the role of RAGE. However, since the S100A8/A9-induced aggravation of VILI was already significantly diminished in TLR4 mutant mice we believe that the influence of RAGE signaling was not major in our model. In the mouse there is no S100A12 and thus it might very well be possible that in the human situation, there is a role for either RAGE or S100A12. Hence, additional research is needed to study both the RAGE axis and S100A12.

The DAMP S100A12 is another member of the S100 family of proteins and human studies indicate that S100A12 levels are elevated during lung inflammation [17]; [19]. Moreover, it was demonstrated that despite similarly elevated levels of S100A8/A9, S100A12 was more increased in ARDS compared to levels in BALF obtained from cystic fibrosis patients [19]. Although this study was limited by the comparison of adult ARDS patients with pediatric cystic fibrosis patients and the fact that BALF was obtained from cystic fibrosis patients during a bronchoscopy performed because of increased respiratory symptoms suggestive of new infection, the difference in expression ratio suggest an important role for S100A12 in the onset of acute neutrophilic lung inflammation [19]. S100A12 can induce ROS, cytokines, and activate RAGE and is therefore considered to be a pro-inflammatory mediator [42]. Again, the function of S100A12 is however difficult to study in murine models since mice do not express S100A12. However, it was recently reported that transgenic mice expressing human S100A12 in a model of allergic pulmonary inflammation did not have increased lung inflammation [43]. These unexpected findings underscore the complexity of the role of S100 proteins and a more pleiotropic role of S100A12 in modulating inflammation was suggested [43]. Whether the lack of S100A12 in mice also influences the role of S100A8/A9 proteins is not known, research studying these proteins should therefore be interpreted with caution.

Increased lung injury may subsequently result in a further rise of S100A8/A9 levels as demonstrated here and it can be hypothesized that this uncontrolled loop of DAMP-mediated inflammation is relevant in ARDS development. Disrupting the S100A8/A9 signaling pathway with S100A8/A9 blocking antibodies would be an attractive approach to attenuate pulmonary inflammation. It has been demonstrated that passive immunization with anti-S100A8 and anti-S100A9 inhibits the accumulation of neutrophils in response to monosodium urate crystals in a murine air-pouch model [36]. In contrast, in a mouse model of lung inflammation induced by LPS inhalation had anti-S100A8 only a weak anti-neutrophil recruitment effect and anti-S100A9 had no effect at all [44]. These results plead against a major therapeutic potential. Others have speculated that the DAMP function of S100A8/A9 proteins is only elucidated in situations with sufficient cell death as S100A8/A9 proteins in the extracellular space function as a danger signal for the immune system [45]. LPS-induced lung inflammation would not have resulted in enough cell death and therefore the antibodies did not function. In line, in a mouse model of streptococcal pneumonia anti-S100A8 and anti-S100A9 caused neutrophil and macrophage recruitment to alveoli to diminish by 70–80% [45]. It has previously been demonstrated that VILI can lead to cell death [4]. Whether these antibodies work in VILI models and have the potential to attenuate the inflammatory response is an interesting subject for further research.

Different studies have indicated that S100A8/A9 proteins also have bactericidal activity [46]–[47]. The innate immune response may herein act as a double edged sword; it is important to fight intruding pathogens, but on the other hand, overwhelming inflammation can cause tissue damage. The presence of S100A8/A9 can severely increase the inflammatory response induced by HV_T_ MV. Future research may study if the reduction in MV-induced injury comes at a cost of an increased susceptibility to live bacterial challenge.

Our study has limitations. First, the longer operation time, clamptime and pumptime of the cardiac surgery patients that developed ALI may also have influenced S100A8/A9 protein levels. For instance, it was previously shown that S100A8/A9 plasma levels are increased during cardiac surgery [48]. In our study S100A8/A9 levels were measured locally in BALF. We believe that this rise is a result of the inflammatory response within the lung and not due to systemic production. Second, since multiple factors may have influenced ALI development in patients a direct comparison between the mouse and the human studies is only partly possible. We used healthy young animals in our murine model and lung injury was solely induced by injurious ventilation combined with LPS-exposure. Third, we restricted our analysis to well-known parameters of lung inflammation and injury. Effects of S100A8/A9 proteins on lung mechanics were not measured.

Taken together, our data clearly demonstrate that S100A8/A9 proteins increase during lung injury and contribute to pulmonary inflammation in a 2-hit setting of HV_T_ MV combined with LPS. Moreover, high levels of S100A8/A9 have synergistic effects with HV_T_ MV, amplifying VILI via TLR4. These results may be translated into novel ARDS therapies in which dysregulated inflammation is attenuated by targeting endogenous S100A8/A9 proteins.

## Supporting Information

Data S1 **Data S1 demonstrate blood pressures and heart rate throughout the experiment.**
(DOC)

Data S2 **Data S2 demonstrate blood gas analysis.**
(DOC)

Data S3 **Data S3 demonstrate PaO_2_/FiO_2_ ratios and outcome parameters of patients with ALI.**
(DOC)

Data S4 **Data S4 demonstrate S100A8 stainings of lungs of S100A9 KO mice.**
(DOC)

Data S5 **Data S5 demonstrate cytokine and chemokine concentrations in lung tissue homogenates.**
(DOC)

Data S6 **Data S6 demonstrate S100A8/A9 levels in mice ventilated with low tidal volume.**
(DOC)
