# Supplementary material for: High Levels of S100A8/A9 Proteins Aggravate Ventilator-Induced Lung Injury via TLR4 Signaling
Source: PLoS One. 2013 Jul 18;8(7):e68694. doi: 10.1371/journal.pone.0068694 (PMC3715539; doi:10.1371/journal.pone.0068694)
Supplement: Data S1 — demonstrate blood pressures and heart rate throughout the experiment. (DOC) [file pone.0068694.s001.doc]

**High levels of S100A8/A9 proteins aggravate**

**ventilator-induced lung injury via TLR4 signaling**

Maria T. Kuipers, Thomas Vogl, Hamid Aslami, Geartsje Jongsma, Elske van den Berg Alexander P.J. Vlaar, Joris J.T.H. Roelofs, Marcus J. Schultz, Nicole P. Juffermans, Tom van der Poll, Johannes Roth, Catharina W. Wieland.

**Online Data supplement**

**Supplemental data S1**

**Blood gas analysis of high tidal ventilated** animals

|  | | **pH** | **PaO_2_** | **PaCO_2_** | **HCO_3_^-^** | **BE** |
| --- | --- | --- | --- | --- | --- | --- |
| **HV_T_ MV** | WT | 7.47 [0.05] | 171.2 [15.2] | 30.2 [4.43] | 20.5 [1.35] | -1.80 [1.37] |
|  | KO | 7.38 [0.02] | 145.7 [13.4] | 39.5 [3.30] | 22.6 [1.32] | -2.05 [1.11] |
| **HV_T_ MV + LPS** | WT | 7.38 [0.05] | 106.8 [12.9] | 48.0 [6.77] | 25.9 [0.71] | 1.89 [1.58] |
|  | KO | 7.43 [0.05] | 136.5 [20.5] | 42.3 [5.21] | 26.3 [1.44] | 0.33 [0.72] |

Data are mean [SEM] of ventilated healthy and LPS-exposed wild-type (WT) and S100A9 knockout (KO) mice. Animals were ventilated for 5 hours, n=8 mice per group. PaO_2_ = partial pressure of arterial oxygen in mmHg; PaCO_2_ = partial pressure of arterial carbon dioxide in mmHg; HCO_3_^–^ = bicarbonate in mmol/l; BE = base excess in mmol/l; HV_T_= high tidal volume

Blood gas analysis of low tidal volume ventilated mice

|  | | **pH** | **PaO_2_** | **PaCO_2_** | **HCO_3_^-^** | **BE** |
| --- | --- | --- | --- | --- | --- | --- |
| **LV_T_ MV** | WT | 7.40 [0.05] | 156.4 [9.38] | 37.3 [4.88 | 21.1 [0.76] | -3.01 [1.10] |
|  | KO | 7.41 [0.03] | 137.6 [17.0] | 38.0 [2.85] | 23.1 [0.78] | -1.11 [1.04] |
| **LV_T_ MV + LPS** | WT | 7.35 [0.05] | 139.0 [12.9] | 46.4 [5.55] | 23.7 [1.06] | -2.06 [1.17] |
|  | KO | 7.35 [0.05] | 111.9 [19.65] | 52.7 [7.43] | 26.5 [1.06] | 0.14 [1.24] |

Data are mean [SEM] of ventilated healthy and LPS-exposed wild-type (WT) and S100A9 knockout (KO) mice. Animals were ventilated for 5 hours, n=8 mice per group. PaO_2_ = partial pressure of arterial oxygen in mmHg; PaCO_2_ = partial pressure of arterial carbon dioxide in mmHg; HCO_3_^–^ = bicarbonate in mmol/l; BE = base excess in mmol/l; LV_T_= low tidal volume
