# Supplementary material for: High Levels of S100A8/A9 Proteins Aggravate Ventilator-Induced Lung Injury via TLR4 Signaling
Source: PLoS One. 2013 Jul 18;8(7):e68694. doi: 10.1371/journal.pone.0068694 (PMC3715539; doi:10.1371/journal.pone.0068694)

**High levels of S100A8/A9 proteins aggravate**

**ventilator-induced lung injury via TLR4 signaling**

Maria T. Kuipers, Thomas Vogl, Hamid Aslami, Geartsje Jongsma, Elske van den Berg Alexander P.J. Vlaar, Joris J.T.H. Roelofs, Marcus J. Schultz, Nicole P. Juffermans, Tom van der Poll, Johannes Roth, Catharina W. Wieland.

**Online Data supplement**

**Supplemental data S2**

**Hemodynamic conditions during high tidal mechanical ventilation.**

Hemodynamic parameters observed during 5 hours of high tidal volume mechanical ventilation (MV). Heart rates (**A**) and arterial blood pressures (BP) (**B**) were measured at 3 time points (T=0, T=2.5, and T=5 hours) in healthy (MV) and LPS-exposed ventilated (LPS + HV_T_ MV) wild-type (WT) and S100A9 knockout (KO) mice. Data represent mean (SD) of n=6-8 mice per group.


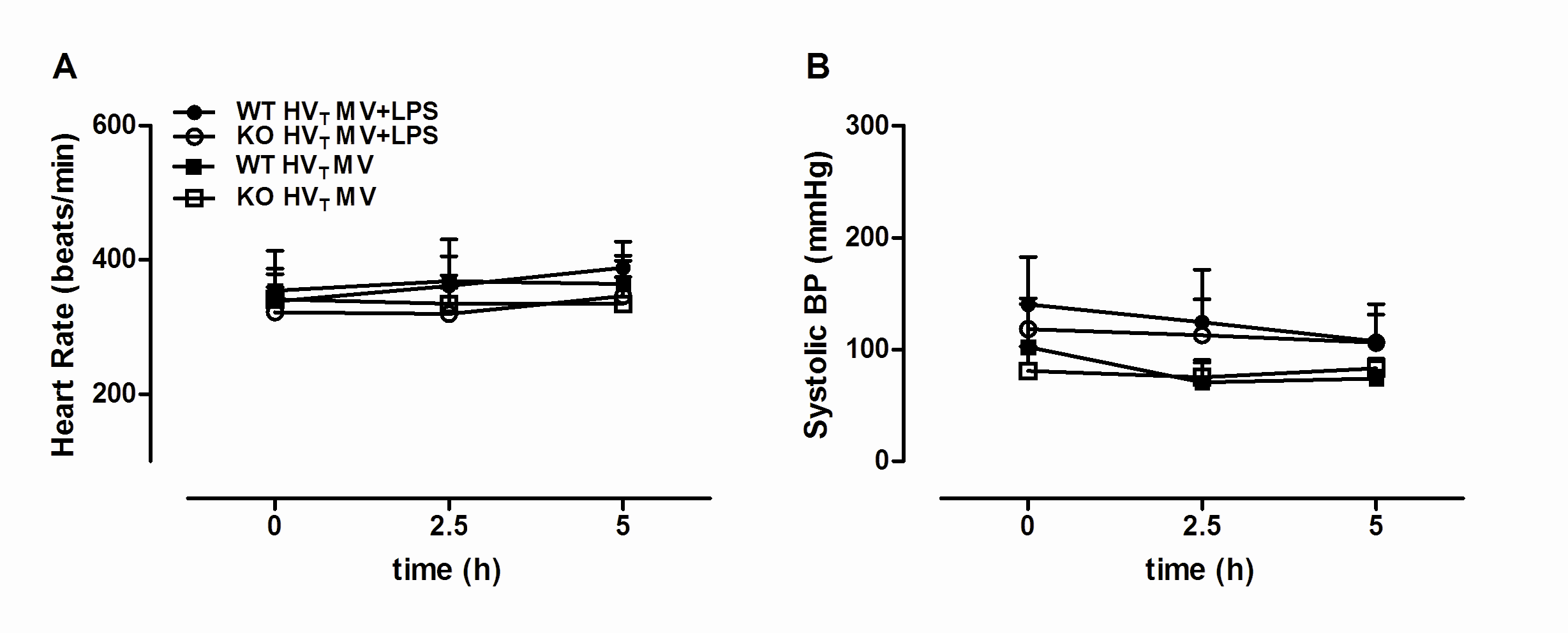


**Hemodynamic conditions during low tidal mechanical ventilation.**

Hemodynamic parameters observed during 5 hours of low tidal volume mechanical ventilation (MV). Heart rates (**A**) and arterial blood pressures (BP) (**B**) were measured at 3 time points (T=0, T=2.5, and T=5 hours) in healthy (MV) and LPS-exposed ventilated (LPS + HV_T_ MV) wild-type (WT) and S100A9 knockout (KO) mice. Data represent mean (SD) of n=8 mice per group.


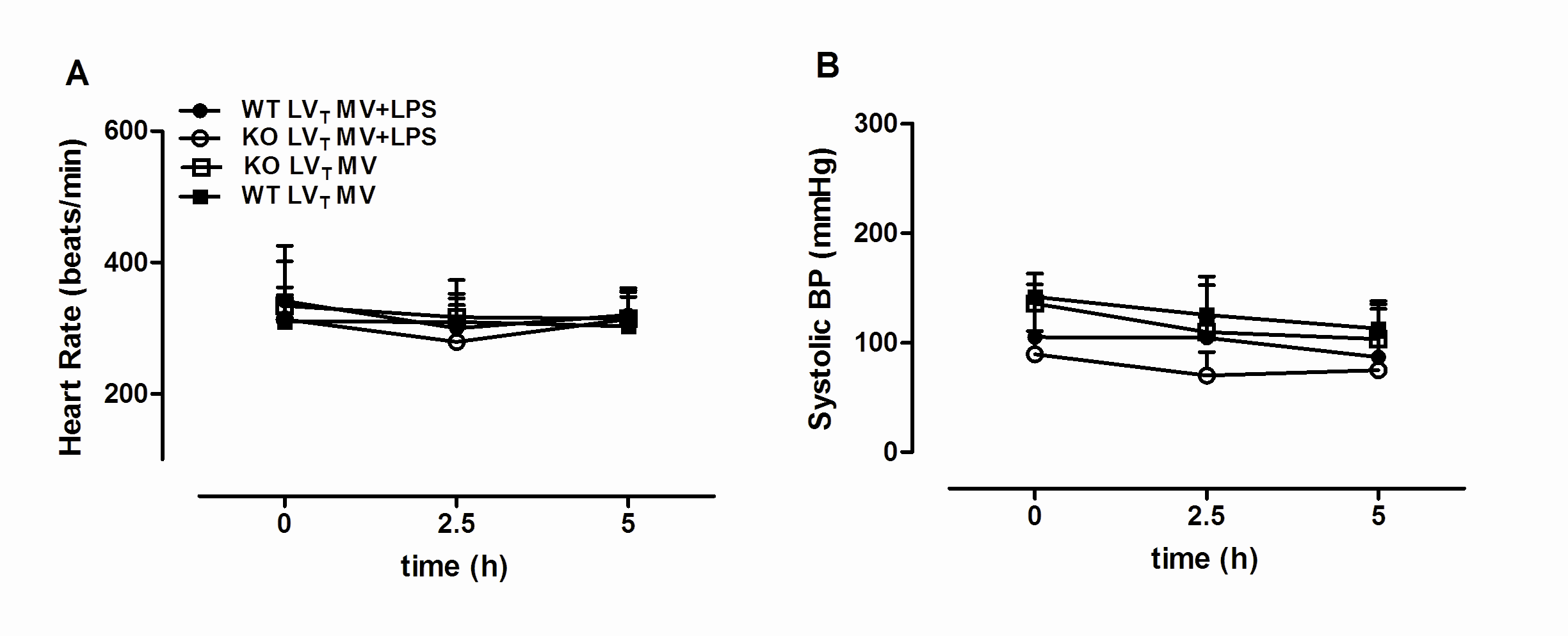

Supplement: Data S2 — demonstrate blood gas analysis. (DOC) [file pone.0068694.s002.doc]
