# Supplementary material for: High Levels of S100A8/A9 Proteins Aggravate Ventilator-Induced Lung Injury via TLR4 Signaling
Source: PLoS One. 2013 Jul 18;8(7):e68694. doi: 10.1371/journal.pone.0068694 (PMC3715539; doi:10.1371/journal.pone.0068694)
Supplement: Data S3 — demonstrate PaO2/FiO2 ratios and outcome parameters of patients with ALI. (DOC) [file pone.0068694.s003.doc]

**High levels of S100A8/A9 proteins aggravate**

**ventilator-induced lung injury via TLR4 signaling**

Maria T. Kuipers, Thomas Vogl, Hamid Aslami, Geartsje Jongsma, Elske van den Berg Alexander P.J. Vlaar, Joris J.T.H. Roelofs, Marcus J. Schultz, Nicole P. Juffermans, Tom van der Poll, Johannes Roth, Catharina W. Wieland.

**Online Data supplement**

**Supplemental data S3**

**Lung injury and outcome characteristics**

|  | **No ALI** | **ALI** |
| --- | --- | --- |
| PaO_2_/FIO_2_ | 176 (145-235) | 114 (98-146)*** |
| Ventilation time (hrs) | 17 (11-24) | 25 (21-83)*** |
| ICU LOS (hrs) | 42 (24-52) | 102 (70-172)*** |
| Hospital LOS (hrs) | 192 (144-252) | 276 (216-594)** |

Acute lung injury (ALI); Intensive care unit (ICU); Length of stay (LOS); data are presented in median (IQR). ***p<0.001, **p<0.01
