# Supplementary material for: High Levels of S100A8/A9 Proteins Aggravate Ventilator-Induced Lung Injury via TLR4 Signaling
Source: PLoS One. 2013 Jul 18;8(7):e68694. doi: 10.1371/journal.pone.0068694 (PMC3715539; doi:10.1371/journal.pone.0068694)

**High levels of S100A8/A9 proteins aggravate**

**ventilator-induced lung injury via TLR4 signaling**

Maria T. Kuipers, Thomas Vogl, Hamid Aslami, Geartsje Jongsma, Elske van den Berg Alexander P.J. Vlaar, Joris J.T.H. Roelofs, Marcus J. Schultz, Nicole P. Juffermans, Tom van der Poll, Johannes Roth, Catharina W. Wieland.

**Online Data supplement**

**Supplemental data S4**

**S100A8 protein staining in S100A9 KO mice.**

Representative images of immunohistochemical pulmonary stainings of S100A8 (specific staining in red, background staining in blue). S100A9 knockout (KO) mice were healthy and spontaneously breathing (C), ventilated with high tidal volume mechanical ventilation (HV_T_ MV), exposed to lipopolysaccharide (LPS), or exposed to LPS followed by high tidal volume mechanical ventilation (HV_T_ MV + LPS) for 5 hours.


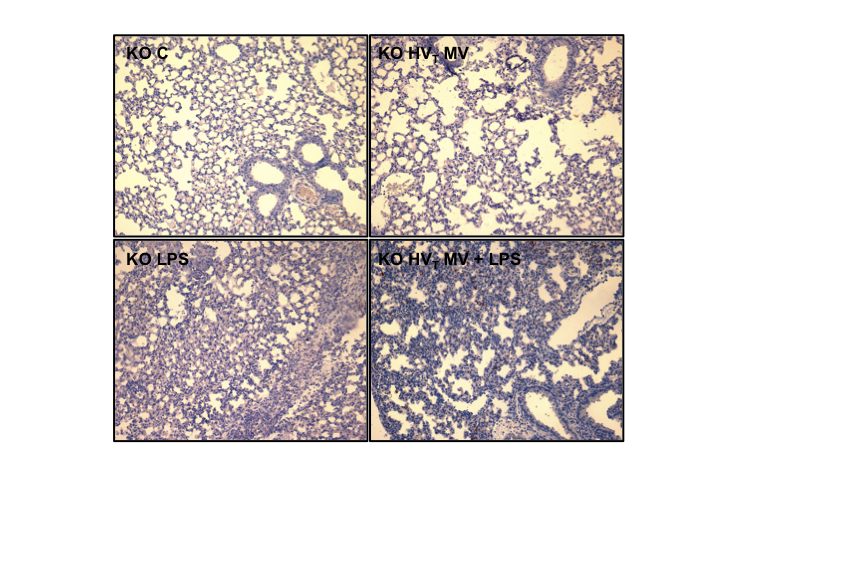

Supplement: Data S4 — demonstrate S100A8 stainings of lungs of S100A9 KO mice. (DOC) [file pone.0068694.s004.doc]
