# Supplementary material for: High Levels of S100A8/A9 Proteins Aggravate Ventilator-Induced Lung Injury via TLR4 Signaling
Source: PLoS One. 2013 Jul 18;8(7):e68694. doi: 10.1371/journal.pone.0068694 (PMC3715539; doi:10.1371/journal.pone.0068694)

**High levels of S100A8/A9 proteins aggravate**

**ventilator-induced lung injury via TLR4 signaling**

Maria T. Kuipers, Thomas Vogl, Hamid Aslami, Geartsje Jongsma, Elske van den Berg Alexander P.J. Vlaar, Joris J.T.H. Roelofs, Marcus J. Schultz, Nicole P. Juffermans, Tom van der Poll, Johannes Roth, Catharina W. Wieland.

**Online Data supplement**

**Supplemental data S5**

**Cytokines and chemokines in lung tissue homogenates**

Cytokine and chemokine concentrations in pulmonary tissue of wild-type (WT) and S100A9 knockout (KO) mice. Animals were spontaneously breathing (C), high tidal mechanically ventilated (HV_T_ MV), exposed to LPS followed by spontaneously breathing (LPS) or exposed to LPS followed by high tidal mechanical ventilation (HV_T_ MV + LPS). Levels of interleukin (IL)–6 (A), macrophage inflammatory protein (MIP)-2 (B), tumor necrosis factor-α (TNF-α) (C), IL-1β (D) and keratinocyte–derived chemokine (KC) (E) were determined. Data represent means (SEM) of 6-7 mice per group. *p<0.05, **p<0.01, ***p<0.001 KO versus WT mice. ###p<0.001, ##p<0.01 and #p<0.05 versus WT C. §§§ p<0.001 and §§ p<0.01 versus LPS-only. +++p<0.001 and ++p<0.01 versus HV_T_ MV-only.


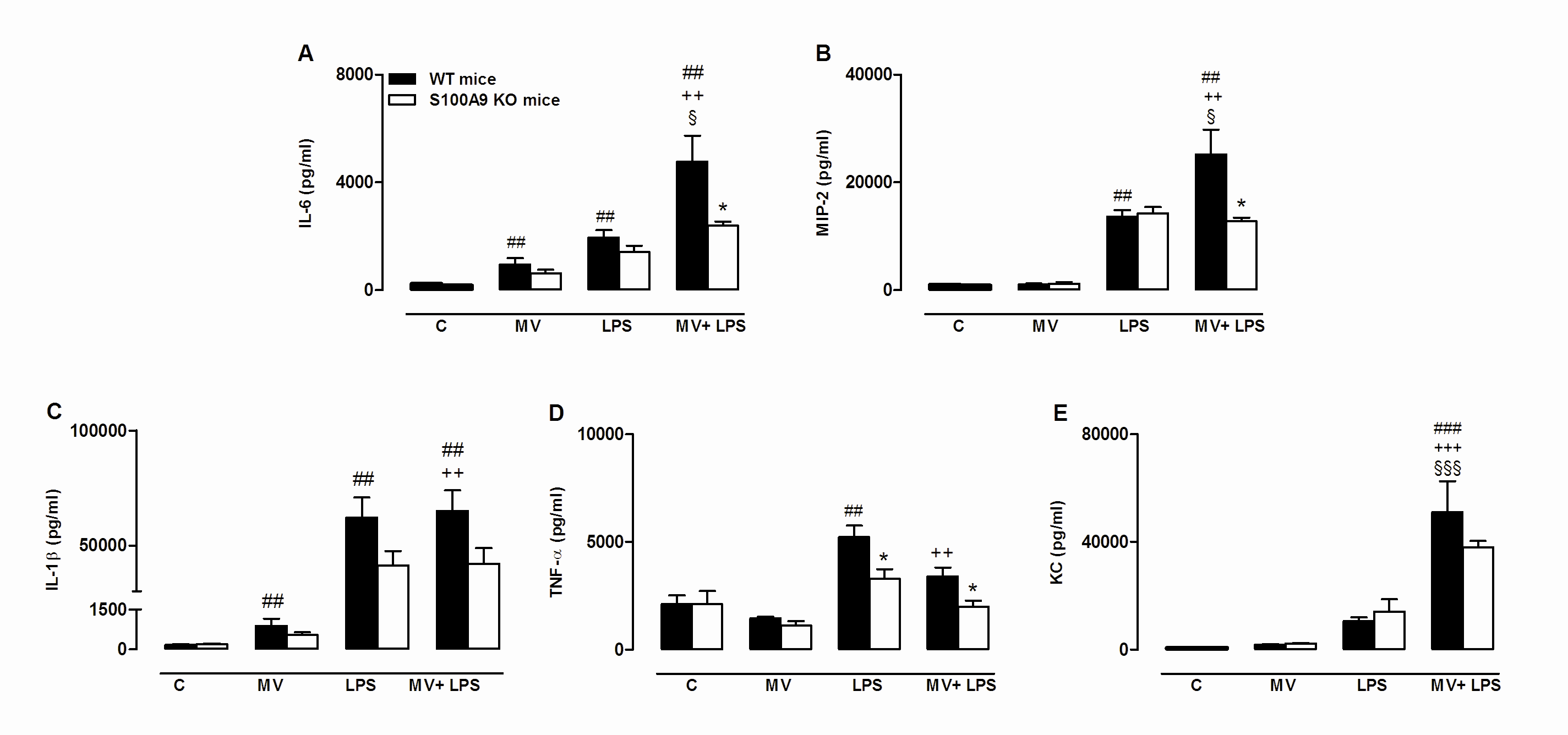

Supplement: Data S5 — demonstrate cytokine and chemokine concentrations in lung tissue homogenates. (DOC) [file pone.0068694.s005.doc]
