# Supplementary material for: High Levels of S100A8/A9 Proteins Aggravate Ventilator-Induced Lung Injury via TLR4 Signaling
Source: PLoS One. 2013 Jul 18;8(7):e68694. doi: 10.1371/journal.pone.0068694 (PMC3715539; doi:10.1371/journal.pone.0068694)

**High levels of S100A8/A9 proteins aggravate**

**ventilator-induced lung injury via TLR4 signaling**

Maria T. Kuipers, Thomas Vogl, Hamid Aslami, Geartsje Jongsma, Elske van den Berg Alexander P.J. Vlaar, Joris J.T.H. Roelofs, Marcus J. Schultz, Nicole P. Juffermans, Tom van der Poll, Johannes Roth, Catharina W. Wieland.

**Online Data supplement**

**Supplemental data S6**

**S100A8/A9 in lung lavage fluid in low tidal volume ventilated mice.**

Levels of S100A8/A9 in lung lavage fluid of low tidal volume ventilated (LV_T_) wild-type (WT) mice with healthy lungs or with pre-existing lung injury induced by LPS-inhalation. Data are shown as mean ± SEM. **P<0.01


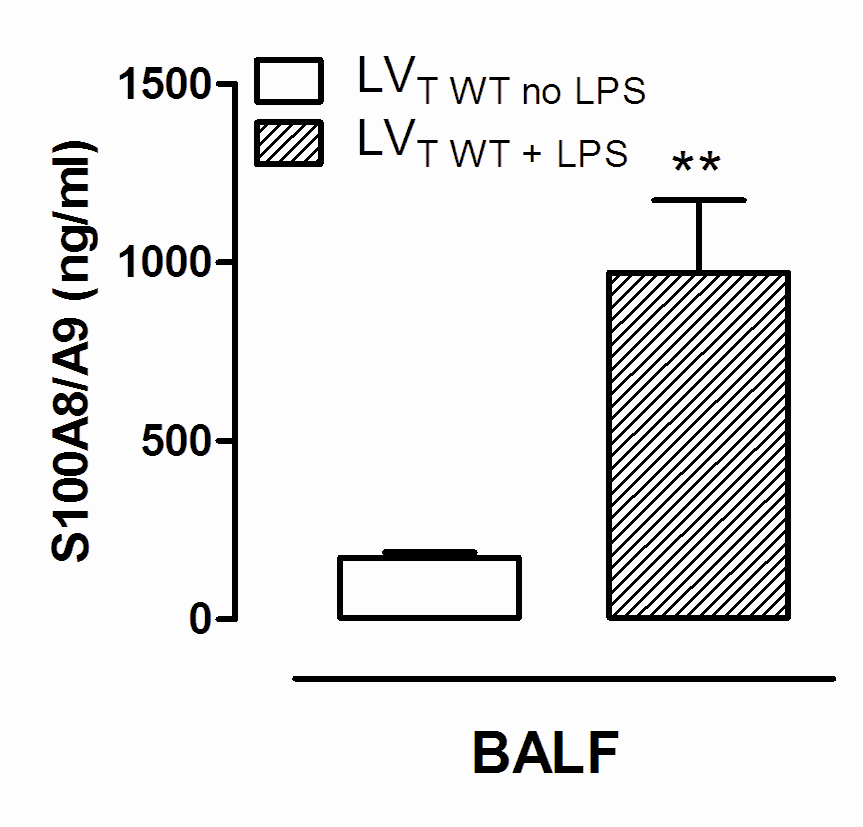

Supplement: Data S6 — demonstrate S100A8/A9 levels in mice ventilated with low tidal volume. (DOC) [file pone.0068694.s006.doc]
